# Neglected kingdoms: the gut virome, mycobiome and their role in inflammatory bowel disease

**DOI:** 10.1080/19490976.2026.2653288

**Published:** 2026-04-02

**Authors:** Yashar Houshyar, Fan Zhang, Paris Tavakoli, Michael C. Grimm, Georgina L. Hold

**Affiliations:** a UNSW Microbiome Research Centre, St George and Sutherland Clinical Campuses, Sydney, NSW, Australia

**Keywords:** Inflammatory bowel disease, Crohn's disease, ulcerative colitis, microbiome, virome, mycobiome

## Abstract

Inflammatory bowel disease (IBD) is a chronic relapsing-remitting disorder of the gastrointestinal tract characterized by immune dysregulation, epithelial barrier dysfunction, and microbial imbalance. While bacterial dysbiosis, including depletion of short-chain fatty acid (SCFA) producers and enrichment of pathobionts, is well characterized, the gut virome and mycobiome remain comparatively neglected. Both exhibit high variability and are constrained by sequencing bias, contamination, and incomplete reference databases, leaving much of the viral and fungal diversity unresolved. Emerging evidence links fungal and viral dysbiosis to IBD pathogenesis, including *Candida* overgrowth, loss of *Saccharomyces*, expansion of Caudoviricetes phages, and detection of eukaryotic viruses such as *Cytomegalovirus* and *Epstein–Barr* virus in inflamed mucosa. These alterations disrupt barrier integrity, modulate immune signaling, and interact with bacteria and archaea in cross-kingdom networks that amplify inflammation. Translationally, the virome and mycobiome are now recognized as therapeutic targets, inspiring interventions from pre/probiotics and synbiotics to precision phage therapy and microbiota-based transplantation, including fecal virome transplantation (FVT) and fecal microbiota transplantation (FMT). This review recognizes the challenges and opportunities of studying these neglected kingdoms, reframes IBD dysbiosis and highlights new directions for biomarker discovery and multikingdom microbiota-directed therapies.

## Introduction

**Figure 1.**
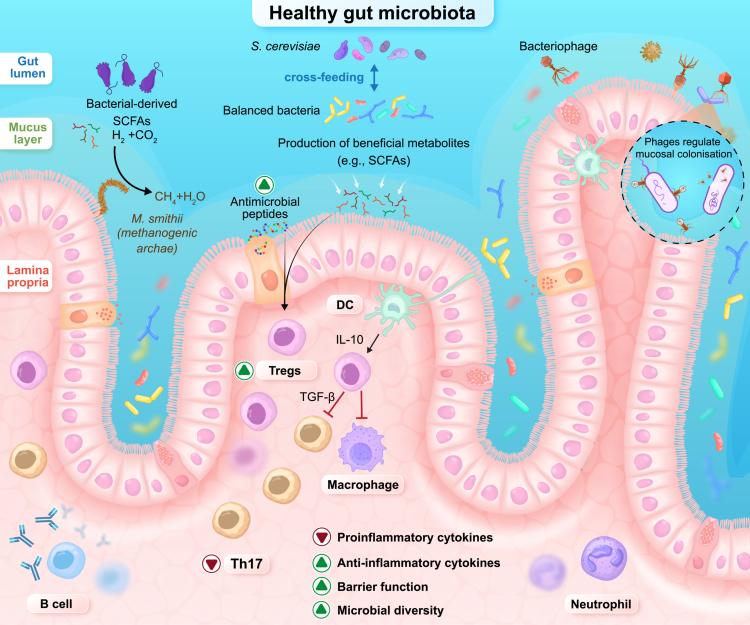
Homeostatic multikingdom interactions in the healthy gut mucosa. The healthy gastrointestinal tract harbors a diverse multikingdom microbiota composed of bacteria, archaea, fungi, and viruses that collectively maintain epithelial barrier integrity and immune tolerance. Balanced bacterial communities ferment dietary substrates to produce short-chain fatty acids (SCFAs), which fuel colonocytes, strengthen tight junctions, and promote regulatory T cell (Treg) differentiation via epigenetic and receptor-mediated pathways, increasing anti-inflammatory cytokines such as interleukin-10 (IL-10) and transforming growth factor-beta (TGF-β). Epithelial antimicrobial peptides (AMPs) support colonization resistance by limiting excessive microbial adherence and preserving spatial organization within the mucus layer. Methanogenic archaea, such as *Methanobrevibacter smithii*, consume fermentation-derived hydrogen, sustaining metabolic cross-feeding and luminal efficiency, while commensal fungi (e.g., *Saccharomyces cerevisiae*) participate in interkingdom metabolic interactions that stabilize microbial networks. Bacteriophages regulate microbial density and mucosal colonization, contributing to ecosystem balance without triggering inflammation. Together, these coordinated host–microbe interactions preserve epithelial barrier integrity and selective permeability, maintaining mucosal homeostasis.

The human gastrointestinal (GI) tract harbors a complex microbial ecosystem of trillions of microorganisms that have colonized from birth and gradually stabilizing into a finely tuned symbiotic community.^1^ The gut microbiota includes diverse entities of bacteria, archaea, fungi, viruses, and protozoa that engage in intricate interkingdom interactions with each other and with the host.^1^ Together, these microorganisms support essential physiological functions such as nutrient metabolism, maintenance of epithelial barrier integrity, antimicrobial peptide (AMP) production; colonization resistance against pathogens; and regulation of host immune responses^2^ (Figure 1).

The dynamic and mutually advantageous relationship between the host and microbiota is crucial for health, whereas environmental, dietary, genetic, and pharmacologic influences can disrupt this balance. Such disruptions lead to intestinal dysbiosis, characterized by loss of microbial diversity and functional changes that predispose to disease.^3^^,^^4^ One condition strongly linked to dysbiosis is inflammatory bowel disease (IBD), encompassing Crohn's disease (CD), and ulcerative colitis (UC).^3^ Despite differences in location and symptoms, both share hallmark features of a dysfunctional mucosal immunity, impaired epithelial barriers, and altered microbial communities.^5^

Research to date has predominantly focused on gut bacteria, which make up over 90% of species, mainly from the Firmicutes, Bacteroidetes, Actinobacteria, and Proteobacteria phyla.^6^ Key genera within these include *Clostridium*, *Eubacterium*, *Ruminococcus*, *Bacteroides*, *Prevotella*, and *Bifidobacterium*, many contributing to fiber fermentation [short-chain fatty acids (SCFAs)], vitamin synthesis (K and B), bile acid transformation, and immune regulation.^6^ SCFAs, particularly butyrate, serve as the primary energy source for colonocytes, reinforce epithelial barrier integrity, promote regulatory T cell (Treg) differentiation through histone deacetylase inhibition, enhance anti-inflammatory cytokine production [including Interleukin-10 (IL-10) and Transforming Growth Factor-beta (TGF-β)], and suppress proinflammatory signaling^6^ (Figure 1). In addition to SCFAs, microbial metabolites such as bile acids play a critical role in maintaining gut homeostasis. Secondary bile acids regulate epithelial integrity, modulate immune responses, and can influence fungal community composition by shaping niche competition and colonization resistance.^1^ In IBD, beneficial bacteria such as *Akkermansia muciniphila* and butyrate-producing species, including *Faecalibacterium prausnitzii* and *Roseburia*, are depleted, leading to reduced SCFAs production, impaired barrier function, and diminished anti-inflammatory signaling.^7^ Concurrently, potentially pathogenic bacteria such as adherent-invasive *Escherichia coli* and *Campylobacter* species often expand, exacerbating inflammation.^8^

Archaea, although less abundant, play significant roles in maintaining gut homeostasis, primarily through methanogenesis. *Methanobrevibacter smithii* consumes hydrogen produced during bacterial fermentation, thereby sustaining efficient short-chain fatty acid production and preventing hydrogen accumulation, which can impair microbial metabolic networks (Figure 1). This species is typically reduced in IBD, potentially disrupting metabolic cross-feeding and luminal fermentation dynamics. In contrast, *Methanosphaera stadtmanae*, which is often enriched in IBD, has been shown to activate dendritic cells (DCs) and induce proinflammatory cytokine production, suggesting direct immune stimulation. These opposing functional shifts underline the importance of examining microbial kingdoms beyond bacteria in understanding IBD pathogenesis.^9^^,^^10^

Notably, the gut virome and mycobiome, which were overshadowed previously, are now gaining recognition as dynamic, disease-associated players in IBD. Their interactions with bacterial and archaeal communities shape microbial networks, regulate the immune response, and improve mucosal health. These insights underscore the need for integrated multikingdom investigations to fully elucidate IBD pathogenesis.^11^

## Fungal and viral microbiota in gut health and IBD

### Gut mycobiome

Although fungi account for less than 0.1% of microbial reads in the gut, their relatively large biomass, complex cell wall structures, and potent immunogenic ligands confer a functionally significant influence on the host–microbe interactions.^12^ The dominant phyla are Ascomycota, Basidiomycota, and Mortierellomycota, with frequently detected genera including *Candida*, *Saccharomyces*, *Penicillium*, *Aspergillus*, *Cryptococcus*, *Malassezia*, *Cladosporium*, *Debaryomyces*, and *Trichosporon*.^12^ However, an ongoing debate remains regarding whether many of these fungi represent stable colonizers or transient, diet-derived passengers, as sampling depth, internal transcribed spacer (ITS) versus 18S ribosomal RNA (rRNA) approaches, and bioinformatic pipelines can markedly influence detection and persistence estimates.^13^

**Figure 2.**
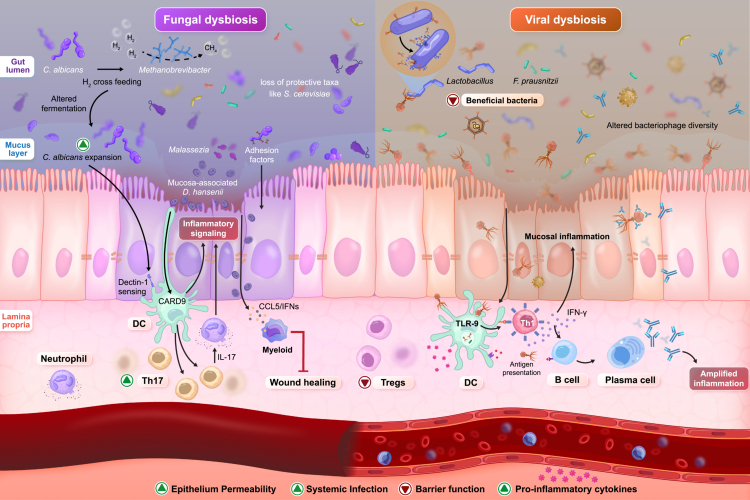
Fungal and viral dysbiosis in IBD. The left panel illustrates fungal dysbiosis in IBD, characterized by the expansion of opportunistic *Candida* species and the loss of protective taxa such as *Saccharomyces cerevisiae*. Fungal adhesion and hyphal-associated factors enhance epithelial interaction and biofilm formation, contributing to oxidative stress and impaired barrier integrity. Fungal cell wall components activate C-type lectin receptors, particularly Dectin-1, triggering caspase-recruitment domain-containing protein 9 (CARD9) signaling and promoting T helper 17 (Th17) responses. Elevated interleukin-17 (IL-17) production drives neutrophil recruitment and amplifies mucosal inflammation. Additionally, mucosa-associated *Debaryomyces hansenii* impairs epithelial wound healing and sustains myeloid-driven inflammatory responses, collectively reinforcing barrier disruption and chronic inflammatory activation. The right panel depicts virome dysbiosis in IBD, characterized by expansion of Caudoviricetes and altered bacteriophage diversity. Phage-derived DNA activates Toll-like receptor 9 (TLR9) on dendritic cells (DCs), promoting T helper 1 (Th1) polarization and interferon gamma (IFN*-*γ) production, thereby amplifying mucosal inflammation. IFN-γ may further modulate B cell differentiation and antibody responses, potentially contributing to immune complex–mediated inflammation. In parallel, stress-induced prophage activation enhances bacterial lysis and lysogenic conversion, promoting epithelial stress and barrier disruption. Together, fungal and viral dysbiosis converge on epithelial barrier disruption, immune amplification, and sustained intestinal inflammation in IBD.

Beyond composition, fungi participate in tightly integrated cross-kingdom and host networks that shape immune regulation and epithelial barrier function.^14^ In experimental models, *Candida albicans* colonization facilitates recolonization of *Bacteroides* following antibiotic exposure and supports epithelial recovery, demonstrating that fungi can indirectly modulate bacterial ecology and mucosal physiology.^15^ Mechanistically, fungal cell-wall components such as β-glucans and mannans engage C-type lectin receptors, particularly Dectin-1, activating the Spleen Tyrosine Kinase (SYK)—caspase recruitment domain-containing protein 9 (CARD9) pathway and downstream inflammasome signaling.^16^ This promotes IL-18 maturation, epithelial restitution, and Interferon gamma (IFN-γ) production in model systems, illustrating how antifungal immunity can sustain barrier integrity.^16^^,^^17^ Conversely, fungal antigens, particularly hyphal-associated adhesion and invasion proteins drive T helper 17 cells (Th17) differentiation and IL-17 secretion. While essential for antifungal defense, exaggerated Th17 responses may amplify chronic inflammation through enhanced IL-17–mediated neutrophil recruitment and tissue damage^17^ (Figure 2).

Diet further modulates the mycobiome composition and immune tone. Plant-rich diets are generally associated with higher fungal diversity and greater representation of non-*Candida* yeasts, whereas Westernized diets favor the expansion of opportunistic *Candida* species and reduced community evenness.^18^^,^^19^ Cross-kingdom syntrophy may link these dietary patterns to mucosal immunity. For example, interactions between *Candida* species and methanogenic archaea such as *Methanobrevibacter* can increase fermentation efficiency and hydrogen turnover, potentially shaping downstream immune signaling^20^^,^^21^ (Figure 2). However, these effects are likely context-dependent, influenced by concurrent bacterial communities and host genotype. CARD9 variants exemplify this gene–environment interplay: loss-of-function alleles impair fungal clearance, reshape fungal–bacterial networks, reduce IL-18 production, and increase colitis susceptibility in humans and animal models.^22^ In addition to these metabolic effects, microbial metabolites such as bile acids may further influence fungal community structure, as alterations in bile acid pools can modulate fungal growth, colonization resistance, and host immune responses.^15^

**Figure 3.**
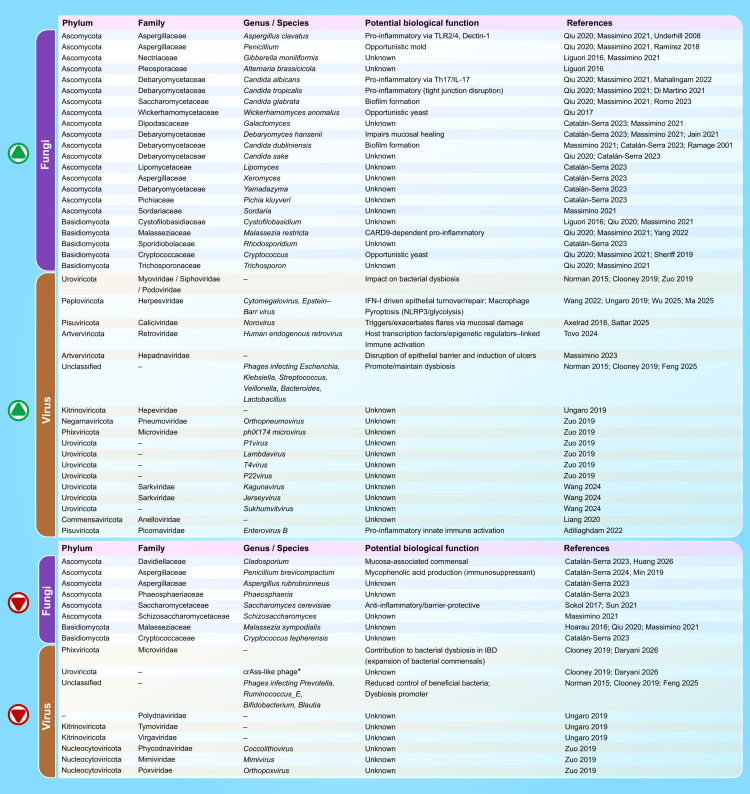
Fungal and viral taxa with altered abundance in IBD and their proposed functional roles. Green upward arrows (↑) indicate increased abundance, whereas red downward arrows (↓) indicate decreased abundance in IBD. Taxa marked with an asterisk (*) represent higher-order taxonomic groups (crAss-like phages corresponding to the order Crassvirales). Abundance changes are referenced in the main text, while additional citations supporting proposed functional roles are provided here where these mechanisms are not explicitly detailed in the manuscript; fungi: *Cladosporium* ^30^, *Aspergillus* ^31^*, Penicillium* ^32^^,^^33^*, Candida* ^34–37^*, S. cerevisiae* ^38^*, D. hansenii* ^27^*, M. restricta* ^39^*, Cryptococcus* ^41^. Viruses: Herpesviridae,^42^^,^^43^ *Norovirus* ^44^, and Picornaviridae ^45.^

In IBD, fungal communities are consistently altered, although causality remains unresolved. Many cohorts report reduced overall fungal diversity, an increased Ascomycota/Basidiomycota ratio, and expansion of *Candida* species, especially *C. albicans* alongside depletion of *Saccharomyces cerevisiae* and certain *Malassezia* species.^23–26^ Additionally, *Debaryomyces hansenii* has been detected in inflamed CD mucosa, where it impairs epithelial wound healing and sustains myeloid-driven inflammatory responses.^27^ Other taxa, such as *Gibberella*, *Alternaria*, *Aspergillus*, and members of Cystofilobasidiaceae, frequently increase,^28^^,^^29^ while Zygomycota lineages decline,^23^ suggesting a shift toward a proinflammatory fungal configuration. These patterns, however, vary by age, disease phenotype, sequencing methodology, antifungal exposure, and biologic therapy, underscoring the need for longitudinal and standardized studies (Figure 3).

Disease activity further refines these signatures. Active disease is associated with the enrichment of opportunistic *Candida* species (e.g., *C. dubliniensis*, *C. lusitaniae*) and a reduction of protective *Saccharomyces* lineages,^23^ and certain fungal shifts correlate with fecal calprotectin (FCP, fecal marker of disease activity) levels; increases in *C. dubliniensis* and *C. lusitaniae* positively correlate with FCP, while *Saccharomyces pastorianus* is negatively correlated, linking specific taxa to the inflammatory burden.^23^ Yet most available studies are cross-sectional and underpowered, making it difficult to distinguish fungi that actively contribute to inflammation from those that expand opportunistically in an inflamed niche.

Emerging mechanistic work highlights the importance of cross-kingdom consortia rather than isolated taxa. *In vitro* and *in vivo* models demonstrate that *C. tropicalis*, together with adherent-invasive *Escherichia coli* (AIEC) and *Serratia marcescens*, form structured biofilms that increase epithelial adherence, increase oxidative stress, exacerbate colitis severity, and impair epithelial barrier integrity.^46^ Network analyses similarly reveal dense fungal–bacterial correlations in healthy individuals who become fragmented in IBD, which is consistent with the breakdown of cooperative interkingdom niches and the emergence of inflammation-promoting biofilm communities.^24^ However, the temporal sequence of these changes and their direct contribution to disease onset remain poorly defined.

Collectively, current evidence supports the view that the gut mycobiome is an active immunomodulatory component of the intestinal ecosystem. Its influence on IBD appears to be mediated through defined host pathways, particularly CARD9–inflammasome–IL-18 signaling and Th17/IL-17 responses, as well as through cross-kingdom biofilm formation and ecological restructuring. Nonetheless, major gaps remain regarding fungal colonization stability, reproducibility of disease-associated signatures and whether targeted manipulation of gut fungi can durably modify the IBD trajectory.

### Gut virome

Following the influence of the fungal community on gut ecology, the virome represents an additional and highly dynamic layer of microbial complexity impacting host immunity and inflammation. The human gut virome comprises viruses that infect eukaryotic cells, bacteria, and archaea.^47^ While eukaryotic viruses contribute directly to host–immune interactions, bacteriophages (phages) that infect bacteria constitute the vast majority of viral sequences detected in the gut and play a central role in shaping bacterial community structure.^48^

Taxonomically, gut phages are dominated by members of the phylum Uroviricota, including classical tailed phages formerly classified as Caudoviricetes (Myoviridae, Siphoviridae, and Podoviridae), alongside Microviridae, Inoviridae, and crAss-like phages.^49^^,^^50^ Many phage lineages mirror the structure of their bacterial hosts; for example, crAss-like phages are strongly associated with Bacteroidetes, whereas Caudoviricetes encompass diverse phages infecting Firmicutes and other dominant gut taxa.^49^^,^^51^ Phages typically exhibit high host specificity, often at the strain level, generating tightly coupled phage–bacteria networks in which viral populations dynamically track bacterial abundance and diversity.^52^ This ecological coupling promotes microbial turnover, niche partitioning, and ecosystem stability under homeostatic conditions.

Functionally, phages operate through two principal life strategies that differentially influence host–microbe dynamics. Lytic phages infect bacterial cells, replicate, and lyse their hosts, thereby contributing to bacterial population control and ecological turnover.^50^ In contrast, temperate phages integrate into the bacterial chromosome as prophages and replicate passively with the host until environmental stress triggers induction. Temperate phages are particularly abundant in the gut and can profoundly influence bacterial fitness through lysogenic conversion, horizontal gene transfer, and the modulation of stress‒response pathways.^50^

Under physiological conditions, the balance between lytic predation and temperate integration supports microbial diversity and ecosystem resilience. In healthy individuals, phages contribute to ecological homeostasis through kill-the-winner dynamics, in which dominant bacterial strains are selectively lysed, preventing competitive exclusion and preserving strain-level diversity.^53^ Lytic phages exert top-down control that limits the expansion of opportunistic pathobionts, while temperate phages may confer adaptive traits that increase bacterial survival without triggering overt inflammation. Together, these complementary mechanisms promote microbial stability, facilitate recovery after perturbation, and help maintain epithelial tolerance.^50^

In IBD, however, this equilibrium appears disrupted. Multiple studies report expansion of Caudoviricetes phages alongside the depletion of the order Crassvirales, a core component of the healthy gut virome.^54^ Whether these alterations represent primary drivers of dysbiosis or secondary consequences of inflammation remains unresolved. Inflammatory stress, antibiotic exposure, and oxidative damage activate the bacterial SOS response, promoting prophage induction and increasing lytic phage production.^55^ This shift from temperate latency to lytic replication may amplify bacterial lysis, release immunostimulatory molecules such as microbial DNA and cell wall fragments, enhance horizontal transfer of virulence genes via lysogenic conversion, and thereby promote epithelial stress and barrier disruption.^55^ Consequently, virome alterations in IBD may reflect a destabilized phage–bacteria network in which stress-induced viral activation reinforces microbial and immune imbalance.

Mechanistically, phages influence inflammation through multiple pathways. In murine models, phage-derived DNA activates Toll-like receptor 9 (TLR9) on dendritic cells, promoting Th1 polarization and IFN-γ production, thereby amplifying proinflammatory mucosal immune activation and exacerbating colitis.^56^^,^^57^ IFN-γ may additionally shape B cell differentiation and antibody responses, potentially contributing to immune complex–mediated inflammation in the chronically inflamed gut. This B cell axis represents a plausible yet incompletely defined extension of phage-driven immune modulation in IBD^58^ (Figure 2). Temperate phages can further intensify dysbiosis by integrating toxin, adhesion, or stress-response genes into pathogenic bacteria such as AIEC, enhancing epithelial invasion and immune activation.^59^ Conversely, depletion of lytic Microviridae may reduce predation pressure on pathobionts,^51^^,^^60^ while loss of phages infecting beneficial bacteria (e.g. *Bifidobacterium*, *Blautia*) may destabilize commensal populations.^59^ These bidirectional phage–bacteria–immune interactions indicate that phages are active modulators of IBD-relevant inflammation rather than passive reflections of bacterial change.

Clinically, virome dysbiosis in IBD commonly features Caudoviricete expansion (including P1-, Lambda-, T4-, Kagunavirus-, and Jerseyvirus-related lineages) alongside depletion of Crassvirales and Microviridae, although patterns vary by disease subtype and sampling site (stool versus mucosa).^54^^,^^61^ However, most studies remain cross-sectional and rely on heterogeneous sequencing and bioinformatic pipelines, and a substantial proportion of viral sequences remain unclassified, limiting functional interpretation and causal inference. Notably, the apparent temporal instability of the gut virome likely reflects both genuine biological dynamics and methodological variability, as differences in viral enrichment, sequencing depth, and bioinformatic pipelines can substantially influence virome detection and interpretation.

Beyond bacteriophages, the eukaryotic virome also exhibits alterations in IBD. Pneumoviridae are increased in UC colonic mucosa,^54^while Anelloviridae prevalence increases with immunosuppressive therapy in IBD patients.^62^ Members of the Herpesviridae family, including *cytomegalovirus* (CMV) and *Epstein–Barr* virus (EBV), are consistently enriched in inflamed tissue in both UC and CD.^63^^,^^64^ Notably, recent longitudinal data indicate that EBV exposure may precede CD development rather than simply reflecting secondary colonization of inflamed tissue. These findings raise the possibility that EBV-mediated immune modulation could contribute to early disease pathogenesis.^65^ Paediatric transcriptomic analyses demonstrate elevated expression of Hepeviridae in CD and Hepadnaviridae in UC.^48^^,^^63^ Furthermore, norovirus increases during CD flares and recapitulates IBD-like pathology through NLR family pyrin domain containing three (NLRP3)/IL-1β activation in mouse models,^66^ while heightened transcriptional activity of human endogenous retroviruses (HERVs) suggests additional host–viral regulatory interactions.^67^ Mouse virome transfer experiments further support causality: healthy human gut viromes protect against colitis in mice, whereas IBD-derived viromes exacerbate inflammation and tissue damage^68^ (Figure 3).

Although the functional consequences of many of these viral shifts remain incompletely defined, the enrichment of pathogenic viral signatures alongside depletion of putative commensals suggests restructuring of the gut virome toward a more proinflammatory configuration in IBD. Whether these alterations initiate disease or represent secondary colonization of inflamed mucosa remains unresolved. Similarly, it is unclear whether Caudoviricete expansion promotes pathobiont selection via lysogenic conversion or whether depletion of taxa such as Crassvirales and Microviridae could serve as reliable biomarkers of disease activity. Addressing these questions will require longitudinal mucosal sampling, standardized sequencing pipelines, integrative multiomics approaches, and mechanistic validation in gnotobiotic and phage-engineering models to disentangle cause from consequence.

### Challenges in detecting the gut microbiome

Early microbiome research depended largely on cultivation techniques, but these methods captured only 10%–30% of gut microorganisms, limiting comprehensive insight into the microbial ecosystem.^69^ The advent of next-generation sequencing (NGS) transformed the field by enabling high-throughput profiling of bacterial communities via 16S rRNA gene sequencing, supported by robust reference databases.^70^ Marker gene analysis remains a fundamental approach for studying microbiome composition and phylogeny.^71^ The rRNA genes, used since Carl Woese's pioneering work, serve well as phylogenetic markers due to their presence in all bacterial and archaeal lineages, combining conserved and variable regions.^71^^,^^72^ However, these advantages do not extend easily to fungi and viruses.

### Detection of the gut mycobiome

Fungi typically constitute less than 0.1% of gut microbial reads, making their detection highly susceptible to contamination and amplification bias.^73^ Their larger, repeat-rich genomes further complicate shotgun metagenomic assembly, which is frequently dominated by bacterial sequences.^74^ Consequently, fungal profiling commonly relies on rRNA markers, particularly the ITS regions (ITS1 and ITS2), flanked by the 18S and 28S rRNA genes. Unlike bacterial 16S rRNA, fungal ITS regions vary substantially in length and sequence composition, enabling species-level discrimination but introducing technical challenges in primer design, amplification efficiency, and read alignment.^75^

While ITS sequencing is considered the primary fungal barcode due to its superior species-level resolution; however, some studies instead employ 18S rRNA gene sequencing. The 18S region provides broader eukaryotic coverage and more conserved primer binding sites, but it offers lower taxonomic resolution for fungi and may amplify nonfungal eukaryotic DNA. Differences between ITS and 18S-based approaches therefore contribute significantly to interstudy variability in reported mycobiome signatures.^76^

Low fungal biomass amplifies both biological and technical distortions. Environmental or dietary fungi (e.g. *Saccharomyces cerevisiae*) may be overrepresented in stool samples, whereas mucosa-associated fungi such as *C. albicans* and *Malassezia restricta* more reliably reflect host-associated communities.^14^ Sampling strategy therefore critically influences interpretation, with biopsies providing greater insight into resident fungal populations than stool-based analyses.^77^ DNA extraction efficiency further contributes to bias, as chitin-rich fungal cell walls are not uniformly lysed by standard kits, potentially underrepresenting specific taxa and skewing relative abundance profiles.^78^

Beyond laboratory constraints, computational factors substantially shape mycobiome interpretation. ITS-based profiling is highly sensitive to region selection (ITS1 versus ITS2), denoising algorithms (e.g. DADA2 versus OTU clustering), trimming thresholds, and chimera removal strategies, which can generate divergent taxonomic outputs from identical raw datasets.^79^ Variation in ITS copy number across fungal species further distorts relative abundance estimates, complicating quantitative comparisons between cohorts and potentially inflating signals for taxa with multicopy rRNA operons.^80^ Incomplete and inconsistently curated reference databases, including limitations within UNITE, restrict taxonomic resolution; clinically relevant taxa such as *Malassezia* species remain underrepresented or inconsistently annotated, contributing to high proportions of unclassified reads in IBD studies.^75^^,^^81^

These analytical variables have direct implications for IBD research. Divergent pipeline choices may partly explain inconsistencies across cohorts reporting *Candida* expansion, stability, or subtype-specific effects. Similarly, the apparent depletion of certain Basidiomycota lineages may reflect database limitations rather than true biological loss. Cross-sectional designs further compound these issues, as low biomass and high interindividual variability reduce statistical power and increase susceptibility to batch effects.^82^

Importantly, cross-kingdom computational integration remains methodologically immature. Most studies analyse fungal data independently of bacterial and viral communities, despite strong ecological interdependence. Compositional data structure, zero inflation, and sparse abundance matrices require advanced statistical approaches, including centered log-ratio transformations, Bayesian hierarchical modeling, and network-based inference frameworks. Multiomics integration strategies capable of simultaneously modeling fungi, bacteria, viruses, host transcriptomics, and metabolomics remain underdeveloped in IBD research.^83^ As a result, interkingdom interactions are often inferred indirectly rather than experimentally validated.

Collectively, these laboratory and computational limitations contribute to variability across IBD mycobiome studies and underscore the need for standardized extraction protocols, harmonized ITS pipelines, improved fungal reference databases, longitudinal sampling, and integrative multikingdom analytic frameworks to enhance reproducibility and mechanistic inference.

### Challenges of viral dark matter

Unlike cellular genomes, viruses exhibit extraordinary genomic diversity, encompassing single- or double-stranded DNA or RNA genomes that may be segmented or non-segmented, linear or circular.^84^ Their rapid replication cycles, high mutation rates driven by error-prone polymerases, and frequent recombination generate extensive sequence divergence, making viruses among the most genetically heterogeneous biological entities.^84^ In contrast to bacteria and fungi, viruses lack universal phylogenetic markers analogous to 16S rRNA or ITS regions, preventing straightforward taxonomic profiling and complicating evolutionary reconstruction.^85^

Traditional culture-based approaches capture only a small fraction of the gut virome due to strict host specificity and limited cultivability, leaving much viral diversity unexplored, the so-called “viral dark matter.”^85^ Culture-independent shotgun metagenomic sequencing has substantially expanded virome discovery; however, viral reads typically constitute less than 5% of total sequencing output, with bacterial and host DNA dominating datasets.^86^ Viral enrichment strategies such as filtration, density-gradient centrifugation, and nuclease treatment improve signal-to-noise ratios but introduce systematic biases, often underrepresenting RNA viruses and temperate phages integrated within bacterial chromosomes.^86^

Computational challenges further constrain interpretation. Incomplete and unevenly curated viral reference databases result in 70%–90% of detected viral sequences remaining unclassified in many IBD cohorts.^87^ Viral genomes are frequently mosaic, shaped by recombination and horizontal gene transfer, which hampers de novo assembly, strain-level resolution, and accurate gene annotation. Assembly fragmentation and short contigs limit confident functional prediction, particularly for auxiliary metabolic genes and virulence factors that may be central to disease mechanisms.^87^

Assigning bacteriophages to their bacterial hosts remains a major bottleneck in mechanistic inference. Current strategies, including CRISPR spacer matching, prophage integration detection, sequence composition similarity, Hi-C proximity ligation, and machine-learning classifiers, provide partial and often low-coverage host predictions.^88^ Inaccurate host linkage complicates interpretation of phage–bacteria networks in IBD and limits causal conclusions regarding whether phage expansions drive pathobiont selection or merely mirror bacterial shifts.

Temporal instability adds another layer of complexity. The gut virome is highly individualized and exhibits substantial short-term variability influenced by diet, infection, antibiotic exposure, and inflammation.^89^ Distinguishing true ecological instability from technical variability (e.g. extraction efficiency, amplification bias, sequencing depth, and bioinformatic pipeline differences) remains challenging. Cross-sectional designs, which dominate current IBD studies, are therefore poorly suited to disentangle cause from consequence. Moreover, stool-based sampling may fail to capture mucosa-associated viruses that interact more directly with epithelial and immune compartments.^90^

RNA viruses represent an additional blind spot. Extraction protocols and DNA-centric library preparation methods bias detection toward DNA viruses, leaving RNA viral communities such as Picobirnaviridae and Astroviridae undercharacterized despite emerging evidence of their immunological relevance.^49^ Extending these assembly challenges, integrated prophages may be missed or misclassified depending on binning strategies.

Importantly, many virome analyses rely on compositional relative abundance data without accounting for absolute viral load, leading to potential misinterpretation of expansion versus proportional shifts. Advanced computational approaches, including long-read sequencing, viral metagenome assembly pipelines (e.g. VIBRANT, VirSorter2, DeepVirFinder), network-based host prediction models, and integrative multiomics frameworks, are beginning to address these gaps but remain inconsistently applied across cohorts.^91^

Collectively, these laboratory, computational, and analytical constraints contribute to substantial variability across IBD virome studies and limit robust causal inference. Standardized extraction protocols, harmonized bioinformatic workflows, expanded reference databases, longitudinal mucosal sampling, and integrative modeling of viral–bacterial–host interactions are essential to move the field beyond association toward mechanistic resolution.

## Clinical and therapeutic applications of gut microbiota modulation in IBD

Therapeutic strategies in IBD have traditionally focused on immunosuppressive and anti-inflammatory drugs, but modulation of the gut microbiota has emerged as a complementary approach aimed at restoring ecological balance and mucosal homeostasis.^92^ Most microbiota-directed interventions have targeted bacterial communities.^93^ Prebiotics such as inulin, fructooligosaccharides (FOS), and galactooligosaccharides (GOS) selectively promote beneficial bacteria, including *Lactobacillus* and *Bifidobacterium*, and some clinical trials report improvements in FCP levels and SCFA production in subsets of UC and CD patients.^94^^,^^95^ However, evidence for consistent endoscopic or histologic remission remains limited and heterogeneous across studies.

Probiotics, including *Bifidobacterium longum*, *Lactobacillus salivarius*, and multistrain formulations, have demonstrated benefits in reducing clinical disease activity and maintaining remission in mild-to-moderate UC. Nonetheless, objective endpoints such as mucosal healing and histologic remission are less consistently achieved, and efficacy appears strain-specific.^96^ Synbiotics combining pre- and probiotics show higher remission rates in some UC cohorts, particularly in patients with longer disease durations, but large-scale standardized trials are lacking.^97^

The antifungal probiotic *Saccharomyces boulardii* has shown benefit in UC and potential in CD through epithelial barrier enhancement and inflammatory modulation.^98^ While clinical improvements are consistent, endoscopic outcomes vary across studies, warranting larger randomized controlled trials (RCTs).

Importantly, although these interventions primarily target bacteria, emerging evidence suggests secondary cross-kingdom effects. Restoration of bacterial diversity may reduce opportunistic fungal expansion (e.g. *Candida*) by limiting niche availability and inflammatory signaling. Similarly, the modulation of bacterial populations may alter phage dynamics, as temperate bacteriophage induction is tightly linked to bacterial stress responses and antibiotic/probiotic pressures.^99^ However, a systematic evaluation of how probiotics, prebiotics, or synbiotics influence fungal or viral dysbiosis in IBD patients remains scarce.

While bacteria-directed therapies demonstrate variable but measurable clinical benefits, their effects on fungal and viral communities remain incompletely understood. Bacteria-targeted interventions may reshape gut fungal and virome compositions, potentially contributing to interindividual variability in treatment response. Expanding microbiota-directed strategies to incorporate multikingdom ecological restoration may therefore provide a more comprehensive approach to IBD management, although targeted fungal and virome-based therapies remain in the early stages of investigation.

### Therapeutic targeting of the gut virome

Alterations in the gut virome are increasingly recognized in IBD, yet the specific viral contributions to microbial dysbiosis and inflammation remain incompletely defined.^100^ Much of the current mechanistic insight derives from controlled preclinical models rather than established clinical interventions.

Human microbiota-associated (HMA) mouse models provide seminal mechanistic evidence for virome-driven effects in IBD. UC-derived viral-like particles (VLPs), which are transferred into bacterially colonized mice prior to dextran sodium sulfate (DSS)-induced colitis, selectively expanded temperate phages, recapitulated UC-associated bacteriome shifts (including *Eubacterium limosum* depletion and *Escherichia–Shigella* expansion), and exacerbated disease severity with heightened cytokine expression independent of whole microbiota transfer. In contrast, healthy donor viromes attenuated colitis in comparable settings, indicating context-dependent phage pathogenicity. Notably, DSS primarily models acute epithelial injury rather than chronic immune-mediated IBD, and therefore translational extrapolation requires caution.^68^

Phage therapy is being revisited as a precision strategy to selectively target pathogenic bacteria while minimizing collateral damage to commensal communities.^101^ In particular, bacteriophages directed against AIEC, a pathobiont implicated in CD, have demonstrated efficacy in reducing bacterial burden and intestinal inflammation in murine ileitis models.^102^ Engineered phages such as HER259, including optimized constructs designed to overcome bacterial resistance or enhance mucosal targeting, represent an emerging avenue of investigation. Nevertheless, these approaches remain preclinical, and key concerns, including immune recognition of phage particles, ecological instability, off-target microbial effects, and long-term safety, remain unresolved.^102^^,^^103^

Fecal virome transplantation (FVT), involving the transfer of purified viral fractions largely enriched with bacteriophages, has been proposed as a strategy to modulate bacterial ecology without transferring whole microbial communities. Preclinical studies suggest that FVT can reshape bacterial networks and influence inflammatory outcomes.^104^ However, human data remain limited and preliminary. Notably, a recent large randomized clinical trial evaluating bacteria-depleted fecal filtrate transfer for recurrent *Clostridioides difficile* infection (rCDI) infection did not demonstrate superiority over placebo, underscoring the challenges of translating filtrate-based or virome-focused interventions into consistent clinical benefits.^105^ Safety concerns, including inadvertent transfer of pathogenic eukaryotic viruses, continue to constrain broader clinical application.^106^

Beyond whole-phage approaches, phage-derived lytic enzymes such as endolysins offer a more targeted antimicrobial strategy. These enzymes can selectively lyse pathogenic bacteria, including *Clostridioides difficile* and *Enterococcus faecalis*, while sparing beneficial taxa.^107^^,^^108^ In experimental models outside the gut, recombinant endolysins have also demonstrated anti-inflammatory effects independent of direct bacteriolysis, suggesting potential immunomodulatory properties.^109^ Whether such effects translate to intestinal inflammation in IBD remains to be determined.

Collectively, current evidence supports a model in which virome-directed therapies have mechanistic plausibility but remain largely in the preclinical or early translational stage. While phage and virome modulation strategies offer theoretical advantages in terms of selectivity and ecological precision, robust clinical trials with longitudinal mucosal sampling, standardized virome characterization, and defined immune endpoints are required before therapeutic implementation can be considered. At present, virome targeting in IBD should be regarded as a promising but still exploratory field.

### Fecal microbiota transplantation (FMT)

Fecal microbiota transplantation (FMT) involves transferring stool-derived microbial communities from healthy donors to recipients, typically via colonoscopy, enema, or oral capsules, with the aim of restoring microbial balance and mucosal homeostasis.^110^ FMT is highly effective for rCDI, where it achieves sustained clinical remission rates exceeding 80%, largely through the restoration of bacterial diversity and the suppression of Proteobacteria overgrowth.^111^

Interest in FMT for IBD emerged following early case reports and RCTs in UC.^112^^,^^113^ Several studies have demonstrated increased rates of clinical remission compared to placebo; however, the effects on endoscopic and histologic remission are more variable and less consistently achieved. Heterogeneity in donor selection, preparation methods, dosing frequency, antibiotic pretreatment, and delivery routes has limited direct comparison between trials.^114^ A recent Australian double-blind study reported clinical remission in 53% of UC patients receiving antibiotic pretreatment followed by oral lyophilized FMT versus 15% with placebo, suggesting that donor profiling and microbial engraftment may critically influence outcomes.^115^ Nonetheless, durable endoscopic healing remains inconsistent across cohorts.

In CD, evidence is more limited and heterogeneous, with small cohorts reporting improvements in clinical indices and steroid-free remission that correlate with successful donor microbiota engraftment.^115^ However, controlled data remain sparse, and reproducibility across populations has not yet been firmly established.

Emerging evidence suggests that FMT may exert cross-kingdom effects beyond bacterial modulation. Virome analyses demonstrate that bacteriophage communities are transferred alongside bacterial taxa and that shifts in phage composition may contribute to ecological restructuring and inflammatory modulation. Similarly, fungal communities are altered post-FMT, although whether these changes directly contribute to therapeutic benefit or represent secondary ecological effects remains unclear. These findings highlight that FMT should be considered a multikingdom intervention rather than purely bacterial therapy.^116^

Recent innovation has focused on standardized capsule formulations, multi-donor pooling strategies, and defined microbial consortia to improve safety, reproducibility, and patient acceptability.^117^ A 2025 international consensus established recommendations for donor screening, preparation protocols, dosing regimens, and follow-up endpoints to increase comparability across trials.^118^

Despite encouraging findings, FMT in IBD remains an evolving therapeutic modality. Response variability, incomplete understanding of mechanisms, uncertainty regarding long-term safety, and inconsistent effects on objective mucosal healing underscore the need for mechanistically guided, longitudinal, multiomics-integrated trials. Future strategies may require refined donor selection based on bacterial, fungal, and viral compositions or the development of targeted microbial consortia rather than whole-stool transfer.

## Future direction

Recent technological advances have substantially expanded our ability to interrogate the gut mycobiome and virome, which were historically constrained by low biomass, lack of universal phylogenetic markers, and incomplete reference databases.^12^^,^^42^ In mycobiome research, ITS-based amplicon sequencing and long-read platforms have improved taxonomic resolution, while curated repositories such as UNITE continue to evolve.^119^ In virome research, viral particle enrichment, shotgun metagenomics, and improved assembly pipelines have expanded the catalog of viral genomes.^120^ Emerging approaches, including long-read sequencing, single-virus genomics, and integrative multiomics analyses, offer promising avenues for resolving strain-level variation and host–virus linkage.^121^

However, several critical barriers remain. A large proportion of viral sequences continue to represent unclassified, limiting confident functional annotation. Detection biases persist, particularly for RNA viruses and integrated prophages.^122^ For fungi, low biomass, ITS copy number variability, and incomplete reference databases complicate quantitative interpretation. Furthermore, cross-kingdom integration remains methodologically immature, with few studies simultaneously modeling bacterial, fungal, viral, and host transcriptomic data in longitudinal designs.^123^ Addressing these analytical and computational gaps is essential before multikingdom therapeutic strategies can be reliably implemented.

Looking forward, microbiome-based precision medicine in IBD will likely require a stepwise, evidence-driven framework rather than rapid translation of emerging technologies. While AI-driven modeling and personalized microbiome modulation (PMM) strategies are conceptually attractive, their feasibility depends on standardized sequencing pipelines, reproducible biomarker validation, and robust mechanistic studies demonstrating causality rather than association.^124^ Current evidence supporting fungal-guided stratification or viral-guided stratification remains preliminary.

Therapeutically, approaches such as phage therapy, fecal virome transplantation (FVT), and refined fecal microbiota transplantation (FMT) represent promising but still evolving strategies. Animal models provide proof-of-concept for virome modulation; however, human clinical data remain limited, and translating findings from animal studies into human applications presents additional challenges requiring careful consideration and strategic planning. Regulatory barriers further complicate implementation. In several jurisdictions, FMT is regulated as a biological product, and concerns regarding pathogen transmission, long-term ecological effects, and donor screening have prompted movement toward defined microbial consortia or standardized live biotherapeutic products. Similar regulatory challenges apply to FVT and engineered phage therapies, particularly regarding safety, manufacturing consistency, and immune reactivity.

Therefore, future progress will depend on longitudinal mucosal sampling, harmonized multiomics pipelines, improved viral and fungal annotation databases, validated host–microbe interaction models, and carefully designed interventional trials with objective endpoints (e.g. endoscopic and histologic remission). Rather than immediate multikingdom precision therapy, the near-term priority lies in establishing reproducible biomarkers, defining causal pathways, and clarifying how bacterial, fungal, and viral networks interact across disease stages.

Collectively, the next phase of research must move from descriptive cataloging toward mechanistic resolution and translational feasibility, ensuring that innovation in sequencing and computational modeling is matched by rigorous validation and regulatory preparedness.
